# Extraskeletal Myxoid Chondrosarcoma: Clinical and Molecular Characteristics and Outcomes of Patients Treated at Two Institutions

**DOI:** 10.3389/fonc.2020.00828

**Published:** 2020-06-16

**Authors:** Benedetta Chiusole, Axel Le Cesne, Marco Rastrelli, Marco Maruzzo, Martina Lorenzi, Rocco Cappellesso, Paolo Del Fiore, Silvia Imbevaro, Marta Sbaraglia, Philippe Terrier, Pietro Ruggieri, Angelo Paolo Dei Tos, Carlo Riccardo Rossi, Vittorina Zagonel, Antonella Brunello

**Affiliations:** ^1^Medical Oncology 1, Istituto Oncologico Veneto IRCCS, Padova, Italy; ^2^Medical Oncology, Insitut Gustave Roussy, Villejuif, Ile-de-France, France; ^3^Surgical Oncology, Istituto Oncologico Veneto IRCCS, Padova, Italy; ^4^Department of Pathology, University of Padua, Padova, Italy; ^5^Accreditation and Aknowledgment Unit, Istituto Oncologico Veneto IRCCS, Padova, Italy; ^6^Department of Biology and Medical Pathology, Institut Gustave Roussy, Villejuif, France; ^7^Department of Orthopedics and Orthopedic Oncology, University of Padua, Padova, Italy; ^8^Department of Surgery, Oncology and Gastroenterology (DISCOG), University of Padova, Padova, Italy

**Keywords:** extrascheletal myxoid chondrosarcoma, anthracycline, NR4A3, drug holiday, chemotherapy (CHT), surgery, trabectedin

## Abstract

**Background:** Extraskeletal myxoid chondrosarcoma (EMC) is a rare subtype of STS, which usually arises in extremities. It carries reciprocal translocations involving the NR4A3 gene. It displays an indolent behavior, but studies with long follow-up showed a high proportion of local and distant recurrences. For patients with progressing metastatic disease anthracycline-based chemotherapy is the standard front-line regimen, though has limited activity. There is some evidence on possible activity of antiangiogenetics.

**Methods:** This is a retrospective study conducted at Istituto Oncologico Veneto and at Institut Gustave Roussy. All patients with a confirmed diagnosis of EMC from January 1980 to December 2018 were extracted from a prospectively maintained database.

**Results:** 59 patients were identified, 37 male (62.7%) and 22 female (37.3%) with a M/F ratio of 1.7/1. We performed molecular analysis in 23 cases, all carried a EWSR1-NR4A3. Out of 49 patients treated with curative intent, 28.6% developed local recurrence and 40.8% patients developed metastases. In patients who had been radically resected (R0) local recurrence occurred in 7.6% of cases and metastases occurred in 15.4% of cases; in patients treated with R1 surgery, rates of relapse were higher. Twenty patients received chemotherapy for metastatic disease; best response was partial response with clinical benefit in 50% of patients. Fourteen patients received a second line of chemotherapy, with 46.1% disease control rate. A drug holiday was proposed to 8 patients with a mean duration of 22.8 months. Median overall survival was 180 months for the study population and 76 months for metastatic patients. No significant prognostic role was found for all studied variables, yet a trend of better survival for complete surgery, location in extremities of primary tumor and solitary lung metastases was observed. Chemotherapy for metastatic disease was negatively associated with survival.

**Conclusion:** In this large retrospective cohort of patients with ECM, location of primary tumor and solitary lung metastases seem to be associated with better survival. Chemotherapy did not impact survival in unselected patients. Further research is necessary in order to identify more active regimens and to provide clinical and molecular factors to select patients that could delay systemic treatment for metastatic disease.

## Introduction

Extraskeletal myxoid chondrosarcoma (EMC) is a very rare sarcoma subtype, which usually arises in the extremities, although it can originate from any anatomic site, and despite the name suggesting a soft-tissue-only location, there are reports of primary ECM of the bone (1–3). First diagnosis occurs commonly in middle age, with a wide age range, and it is more frequent in men than in women (1, 2, 4–6). It is currently classified by the World Health Organization classification under the category of tumors of uncertain differentiation (7).

EMC was first recognized in 1953 by Stout and Verner, but it was only in 1972 that Enzinger defined precisely its clinical and pathologic features (8). Importantly, ECM harbors recurrent genetic rearrangements involving the *NR4A3* gene on chromosome 9, representing an extremely useful confirmatory diagnostic clue (9) *NR4A3* fuses with different partners. The most frequent is *EWSR1* (EWS RNA-binding protein (1) followed by *TAF15* [TAF 15 RNA polymerase II, TATA box binding protein (TBP)-associated factor]. Rare fusion transcripts have been described, which are TCF12-NR4A3, TFG-NR4A3, and HSPA8-NR4A3 (4, 5, 10, 11).

EMC is considered a disease with an indolent behavior characterized by slow growth, but studies with adequately long follow-up point at a high proportion of local and distant recurrences. In retrospective series, the extension of surgery appears to affect the recurrence rate (1).

Metastases more frequently occur in lung, followed by bone, lymph nodes, and soft tissue. Despite a high rate of metastases, patients typically exhibit remarkably high survival rates of approximately over 80% at 5 years and over 60% at 10 years (1, 2, 6).

At present, no predictive factor is available to help decision-making for metastatic disease, and in particular to define whether systemic treatment should be used. Standard anthracycline-based chemotherapy, which is commonly used in the first-line treatment of advanced soft tissue sarcoma, has limited activity in this sarcoma subtype with variable reported response rates (12). There is some evidence on the role of anti-angiogenics in EMC. An Italian study reported activity of sunitinib on 10 patients treated with sunitinib at the dose of 37.5 mg/day, with best response being partial response in 6 patients and stable disease in 2 patients (13). More recently a multicenter phase 2 study tested the activity of pazopanib, with partial response observed in 4 out of 22 eligible patients (18%) and stable disease in 16 patients (73%) (14).

## Methods

All consecutive patients with a confirmed diagnosis of EMC treated at Istituto Oncologico Veneto in Padova and at Institut Gustave Roussy in Villejuif from January 1980 to December 2018 were extracted from a prospectively maintained database. Electronic health records were reviewed and the following data were collected: date of diagnosis, age at diagnosis, pathology report, performance status, site of primary tumor, site of metastases, first treatment approach, quality of surgery, local recurrence, data on radiation therapy, response to treatment, and survival. Response to treatment was evaluated by means of RECIST criteria, version 1.1 for all patients with metastatic measurable disease. The clinical outcome of each patient was recorded as alive or dead as of November 30, 2019.

Overall survival was measured from different time points (from first diagnosis, from the diagnosis of metastatic disease, and from the start of chemotherapy) to date of death; patients lost at follow-up were censored at last follow-up visit. Statistical analysis was carried out with R version 3.6.1. Survival was estimated with the Kaplan–Meier product-limit method; comparisons between groups were performed using the log-rank test.

The analysis for NR4A3 transcript was performed with qRT-PCR assay: total RNA was extracted from 10 sections of FFPE tissue with manual RNeasy FFPE Kit (Qiagen) and was quantified using a spectrophotometer. cDNAs were synthesized from 1,000 ng of RNA by a reverse transcription, using two reverse primers, respectively, in the exon 2 and in the exon 3 of NR4A3 gene. Three microliters of each cDNA was used in a real-time PCR assay. Primers and probes used in this assay are specific for the detection of the following fusion: EWSR1(ex7)/NR4A3(ex2), EWSR1(ex12)/NR4A3(ex3), EWSR1(ex13)/NR4A3(ex3), and TAF15(ex6)/NR4A3(ex3).

## Results

### Patients' Characteristics

A total of 59 patients were identified, 37 were male (62.7%) and 22 were female (37.3%) with a male-to-female ratio of 1.7/1. Median age at diagnosis was 54 years (range, 24–90 years). Patients' characteristics are described in Table 1.

**Table 1:** Patients' characteristics.

**Patients characteristics**	**N. (%)**
Male	37 (62.7%)
Female	22 (37.3%)
Age	24–90 years
Primary Location	(n. 59)
lower limb	40 (67.8%)
upper limb	6 (10%)
chest	3 (5.1%)
abdomen	7 (12%)
other	3 (5.1%)
Metastatic Sites	(n. 26)
Lung	23 (88%)
Bone	4 (15.4%)
Other	14 (53.8%)
Primary Treatment	(n. 59)
Surgery	42 (71.2%)
Chemotherapy	10 (16.9%)
Radiation therapy	3 (5%)
NA	4 (6.9%)
Extension of Surgery	(n. 49)
R0	26 (53%)
R1	12 (24.5%)
R2	2 (4%)
NA	9 (18.5%)
First Line of Chemotherapy	(n. 20)
Anthracycline-based	11 (55%)
Oral cyclophosphamide	4 (20%)
Other regimens	5 (25%)
Second Line of Chemotherapy	(n. 14)
Anthracycline-based	3 (21.4%)
Trabectedin	3 (21.4%)
Other regimens	8 (57.2%)
Locoregional Treatment	
Radiation therapy	23 (48.9%)
Lung metastasectomy	8 (17%)
Excision of local recurrence	14 (29.8%)
Radiofrequency	2 (4.3%)

Primary tumor site was lower limbs in 40 patients (67.8%), abdominal wall in 7 patients (12%), upper limbs in 6 patients (10%), chest in 3 patients (5.1%), and other sites in 3 patients (i.e., vulva, heart) (5.1%). Location was not available in four patients (6.9%). Median tumor size was 10 cm (range, 1.5–25 cm).

Molecular analysis was performed in 23 patients, detecting the presence of *EWSR1–NR4A3* fusion in all cases.

Fifty-three patients presented with localized disease and six patients were metastatic at diagnosis. Out of 49 patients treated with curative intent, 20 patients (40.8%) developed metastases and 14 patients (28.6%) developed local recurrence.

Most frequent metastatic site was the lung (22 patients); 4 patients had bone metastases and 12 patients presented metastases in other sites (lymph nodes, soft tissue).

### Treatment Description

The first treatment was surgery for 42 patients, chemotherapy for 10 patients, and radiation therapy for 3 patients. Of the patients treated with chemotherapy, seven received treatment in the pre-operative setting.

Data on extension surgery were available for 40 patients: 26 had radical (R0) surgery, 12 patients had surgery with microscopic margin infiltration (R1), and 2 patients had macroscopic presence of tumor (R2).

Among patients with R0 surgery, two had local recurrence (7.6%) and four developed metastatic disease (15.4%); among patients with R1 surgery, five had local recurrence (41.6%) and seven developed metastatic disease (58.3%); of the two patients with R2 resection, one was metastatic and did not undergo further surgery, and the other one did not experience local recurrence after re-excision and radiation therapy. Outcome of surgery is described in Table 2.

**Table 2:** Outcome of surgery.

	**N. (%)**	**N. (%)**	**N. (%)**
**Type of resection**	**Total**	**Local recurrence**	**Metastases**
**R0**	26	2 (7.6%)	4 (15.2%)
**R1**	12	5 (41.6%)	7 (58.3%)
**R2**	2	NA	NA

Twenty patients received chemotherapy for metastatic disease, with 11 patients receiving an anthracycline-based regimen (4 patients received doxorubicin alone and 7 patients received a combination regimen). For 10 evaluable patients treated in first-line setting, best response was partial response in one case and stable disease in five cases with an overall control rate of 60%. Four patients received oral cyclophosphamide obtaining stable disease as best response in one case (control rate 25%); five patients received other regimens (i.e., etoposide, trabectedin), with two patients experiencing stable disease and two patients experiencing progression as best response. Control rate with chemotherapy of all assessable patients was 50%.

Fourteen patients received second-line chemotherapy, which, in three cases, was an anthracycline-based regimen, and all experienced progressive disease as best response; three patients received trabectedin, with two patients experiencing stable disease as best response; pazopanib was administered to one patient with stable disease as best response; other regimens (i.e., etoposide, cyclophosphamide) were used in seven cases, obtaining stable disease in two patients and progression in all other patients as best response. Control rate with second-line chemotherapy in assessable patients was 46.1%.

Among the 20 patients treated in the first-line setting, 6 patients were treated in pre-targeted therapy period (i.e., trabectedin; anti-angiogenics). Data on systemic treatment are reported in Table 3.

**Table 3:** Disease control-rate with of chemotherapy.

**Line of Treatment**	**N. (%)**	**Control Rate**
**First-Line**	20	50%
Anthracycline-based	11	60%
Oral cyclophosphamide	4	25%
Other regimens	5	50%
**Second-Line**	14	46.1%
Anthracycline-based	3	0%
Trabectedin	3	66%
Other regimens	7	28.5%
Pazopanib	1	100%

Seventeen patients received loco-regional therapy, which was radiation therapy in 23 cases, pulmonary metastasectomy in 8 cases, excision of local recurrence in 14 cases, and radiofrequency ablation in 2 cases, with a wide range of number of treatments per patients (from 1 to 17).

A drug holiday was proposed to eight patients with a mean duration of the therapeutic break of 22.8 months (range, 2–41 months), and with two patients still being observed at the time of the writing of this manuscript.

### Survival Analysis

Out of 59 patients, data for 4 patients were not available either because they were seen just once for second opinion or because they were lost at follow-up. With a median follow-up time of 72 months, 20 patients have died.

For the entire group of patients, median OS (mOS) was 180 months, with 75% of patients being alive at 5 years and 63% of patients being alive at 10 years. Considering only patients with metastatic disease, median OS was 76 months.

Median time from diagnosis to metastatic disease was 5.9 years with a proportion of 40.8% of patients treated with curative intent developing metastatic disease.

**Figure 1:**
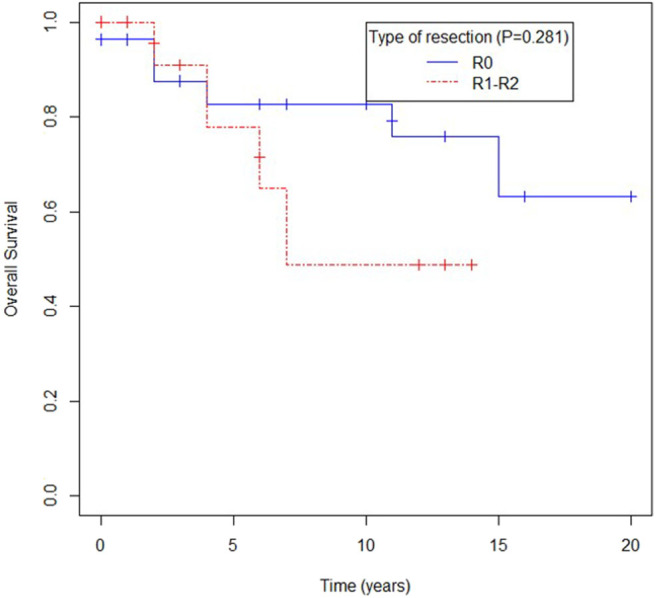
Overall survival according to extention of primary resection.

Extension of primary surgery seemed to impact overall survival, with patients with R0 surgery having a trend toward better survival than patients with R1 and R2 surgery, as shown in Figure 1. The presence of local recurrence did not affect survival (*p* = 0.54).

**Figure 2:**
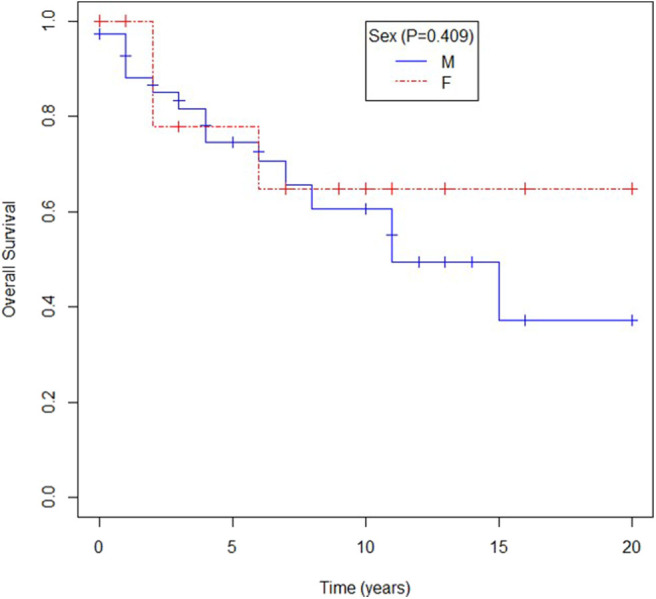
Overall survival according to sex.

No difference in survival rates was observed according to gender (mOS not achieved for female vs. 136 months for male patients *p* = 0.409) as shown in Figure 2.

**Figure 3:**
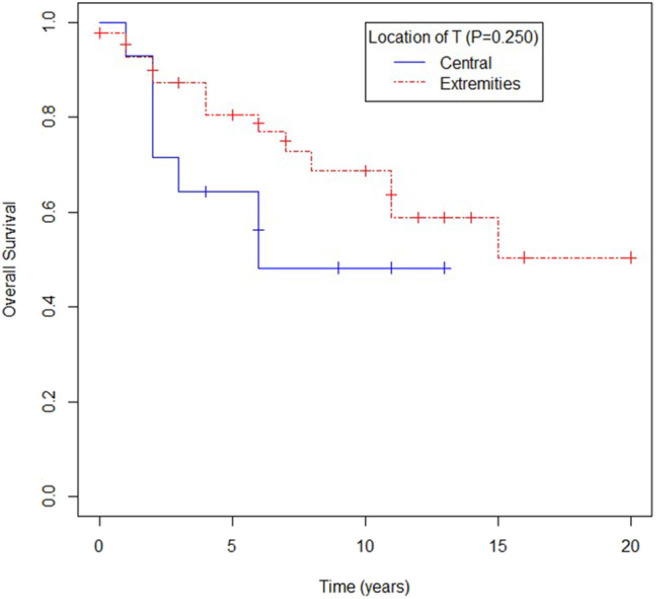
Overall survival according to location of primary tumor (T): central (visceral, trunk, head and neck) or extremities.

Overall survival for patients with primary location in extremities seemed to be better when compared to other primary sites, yet no statistical difference was observed (mOS 180 vs. 73 months; *p* = 0.250) as shown in Figure 3.

**Figure 4:**
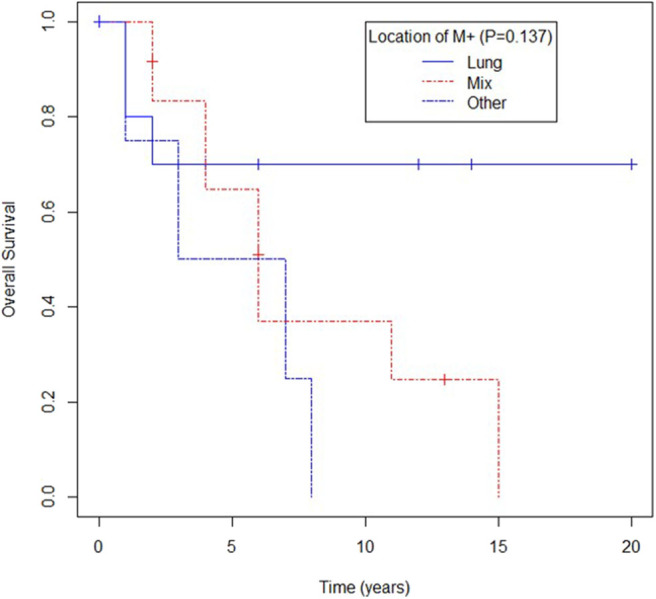
Overall survival according to location of metastases (M) in lung, mixed (lung and other) or other.

Location of metastases in the lung trended toward better survival compared to location in other sites, yet again no statistical difference was observed (mOS for lung metastases not reached, mOS for patients with lung and other sites' metastases being 73 months, and mOS for patients with metastases only in extrapulmonary sites 62 months, *p* = 0.137) as shown in Figure 4.

Chemotherapy in patients with metastatic disease was significantly associated with worse survival (mOS 72 vs. 81 months, *p* = 0.009). Median progression-free survival for patients receiving first-line chemotherapy was 9 months. No predictive role of the studied variables was observed for progression-free survival.

Univariate analysis showed metastases other than lung or mixed, and administration of chemotherapy for metastatic disease being associated with worse survival, with metastatic sites retaining prognostic significance as independent risk factor for survival in multivariate analysis (Table 4).

**Table 4:** Univariate Cox analysis for risk factors.

	**Univariate Cox regression analysis**	
**Characteristic**	**HR (95% CI)**	***p*****-value**
**Gender**		
Male	Reference	
Female	0.686 (0.263–1.786)	0.440
**Age**		
For each 1-year increase	1.009 (0.979–1.041)	0.554
**Primary location**		
Central	Reference	
Extremities	0.567 (0.222–1.446)	0.235
**Location of metastases**		
None	Reference	
Lung	2.856 (0.572–14.250)	0.201
Mixed	6.665 (1.779–24.970)	**0.005**
Other	11.431 (2.465–53.000)	**0.002**
**Type of resection**		
R0	Reference	
R1-R2	2.021 (0.540–7.570)	0.296
**Palliative chemotherapy**		
No	Reference	
Yes	3.856 (1.349–11.020)	**0.012**

*HR, hazard ratio; CI, confidence interval. p value significance are indicated in bold*.

No difference in survival was observed between patients being treated for metastatic disease before targeted therapy era and those treated after (mOS 72 months vs. mOS NA, *p* = 0.59).

## Discussion

This study provides to date one of the largest series of an ultra-rare soft tissue sarcoma subtype with molecular data.

Consistent with previous reports, this study showed a predominance of the disease in male patients. EMC can occur at any age, but most patients in reported series are in the fifth and sixth decade (1, 2, 5, 6), as in our series in which median age at diagnosis is 56 years.

Our study also confirms the higher incidence of primary location in lower limb, which was observed in previous reports, where the most frequent site is thigh followed by upper arm and girdles, with only 10% of tumors arising in chest wall, abdomen, or other sites (1, 2, 4, 5, 15).

As for the role of surgery, there is extensive literature showing that incomplete or marginal surgery in patients with soft tissue sarcoma is associated with high rate of recurrence and metastases (16, 17). In a retrospective series of 117 patients with EMC treated with surgery as primary intent, a rate of 48% of local recurrence and 46% of metastatic recurrence was reported, with extension of surgery not identified as independent risk factor (18). In another surgical series on 87 patients, with data of quality of surgery available for 43 patients, a higher rate of local and metastatic recurrence was observed for patients receiving marginal surgery (1). Data from our study showed a correlation between extension of surgery and rate of local and metastatic recurrence and also a trend of better survival for patients receiving R0 surgery, in accordance to overall data for unselected histological type of soft tissue sarcoma (19–21).

Our study confirms that EMC's behavior is that of an indolent tumor, with most patients having very long survival rates even in the presence of metastatic disease.

Though the presence of distant metastases is an independent adverse risk factor, in our study, we identified patients with solely lung metastases as a subgroup with a better survival at univariate and multivariate analysis, with a proportion of 70% of patients alive at 10 years (1, 15, 18).

Survival rates for primary tumors with central localization were slightly worse than other primary sites, reinforcing evidence deriving from other EMC retrospective series ^18^ and from a large case series that analyzed causes of death in patients with low-grade sarcomas (22).

As for the role of chemotherapy in advanced disease, in our study, standard anthracycline-based chemotherapy was not associated with better survival and, on the contrary, use of chemotherapy appeared to be associated with worse survival. Indeed, anthracycline-based regimens when used as first-line treatment showed a disease control rate of 60%, which is consistent with a previous retrospective study (12) and showed little or no benefit in second-line treatment, with no data on responses ever reported in further lines for this ultra-rare histotype. Other regimens used in first line showed lower control rate, with an overall control rate from other chemotherapy regimens in the range of 50% with no complete responses and low rates of partial response, confirming data of literature (1). Due to the retrospective nature of the study, no definitive conclusion can be drawn on the role of chemotherapy, and the negative impact of chemotherapy on survival could as well be biased by a higher likelihood to propose chemotherapy to patients with higher tumor burden or who are highly symptomatic, therefore having a worse prognosis independent of chemotherapy.

Trabectedin used as second-line regimen achieved a disease control rate of 66%; this is consistent with the only data reported of use of Trabectedin in EMC to date. In a subgroup of patients with diagnosis of EMC treated in the phase II trial of trabectedin, two patients achieved stable disease as best response (23). Filannino et al. (24) described a good response to trabectedin associated to radiation therapy showing synergy.

In our study, two patients received an angiogenesis inhibitor in second line and third line of treatment; both achieved stable disease as best response.

In our study, we observed a progression-free survival time of 9 months, which is higher than what was reported by Drillon et al. in 2008 in 21 patients (5.2 months) and consistent with data reported in 2013 on the use of anthracyclines in 11 patients in the series by Stacchiotti et al. (12, 14) (8 months), but shorter than median progression-free survival achieved with Pazopanib in a recent phase II trial that enrolled 23 patients (19 months) (1).

Again, given the limitations of retrospective data, data on progression-free survival time can be biased by different timing of restaging scans.

Of note, our study is the first to our knowledge to provide data on drug holiday, with long intervals of chemotherapy-free time for eight patients (mean duration of drug-free interval 22.8 months), suggesting the safety of such practice.

No analysis could be made to take into account the type of molecular alteration given the fact that all patients carried an EWSR1-NR3A4 translocated EMC.

## Conclusion

Our study provides clinical and molecular data from one of the largest series of an ultrarare soft tissue sarcoma subtype.

Our data could not suggest any definitive role for quality of surgery of primary tumor, though radical surgery is associated to lower rates of local and metastatic relapse, while showing that location of primary tumor and solitary lung metastases can be prognostic for better survival.

Furthermore, our study adds evidence to the poor performance of anthracycline-based chemotherapy, which was not associated with better outcomes, yet the use of trabectedin translated in overall fair disease control rates.

Our data also suggest the safety of including drug holidays in the treatment strategy of metastatic disease.

Further research is necessary in order to identify more active regimens and to provide clinical and molecular factors to select patients that could delay or even avoid systemic treatment for metastatic disease.

## Data Availability Statement

All datasets generated for this study are included in the article/supplementary material.

## Ethics Statement

The studies involving human participants were reviewed and approved by Comitato Etico of Istituto Oncologico Veneto. The patients/participants provided written informed consent to participate in this study.

## Author Contributions

BC drafting the work, substantial contributions to the acquisition, analysis, and interpretation of data. AL, MR, MM, ML, RC, PD, SI, MS, PT, PR, AD, CR, and VZ substantial contributions to the acquisition, analysis, and interpretation of data. AB revising the work critically for important intellectual content, contributions to the acquisition, analysis, and interpretation of data.

## Conflict of Interest

The authors declare that the research was conducted in the absence of any commercial or financial relationships that could be construed as a potential conflict of interest.
